# The interaction between smoking and bladder cancer genetic variants on urothelial cancer risk by disease aggressiveness

**DOI:** 10.1002/cam4.4654

**Published:** 2022-03-13

**Authors:** Stanley Teleka, Sylvia H. J. Jochems, Karin Jirström, Tanja Stocks

**Affiliations:** ^1^ Department of Surgical Sciences Uppsala University Uppsala Sweden; ^2^ Department of Clinical Sciences Lund Lund University Lund Sweden

**Keywords:** cohort study, genetic risk, interaction, smoking, urothelial cancer

## Abstract

**Background:**

Smoking has shown interactions with bladder cancer (BC) genetic variants, especially N‐acetyltransferase‐2 (*NAT2*), a tobacco smoke metabolism gene, on BC risk. The interactions by disease aggressiveness are unknown.

**Methods:**

We investigated the interaction between smoking and 18 single nucleotide polymorphisms (SNPs) for BC, individually and in a genetic risk score (GRS), on urothelial cancer (UC) risk including BC. We analysed data from 25,453 individuals with 520 incident UCs during follow‐up, 339 non‐aggressive (non‐fatal, non‐muscle invasive) and 163 aggressive (all other) UCs. Hazard ratios (HRs), absolute risks and additive and multiplicative interactions for two‐by‐two combinations of never/ever smoking with low/high genetic risk were calculated.

**Results:**

Smoking and *NAT2* rs1495741 interacted strongly, positively on aggressive UC on both the multiplicative (*p* = 0.004) and additive (*p* = 0.0002) scale, which was not observed for non‐aggressive UC (*p*_interactions_ ≥ 0.6). This manifested in a higher HR of aggressive UC by ever smoking for the slow acetylation *NAT2* genotype (HR, 5.00 [95% confidence interval, 2.67–9.38]) than for intermediate/fast acetylation *NAT2* (HR, 1.50 [0.83–2.71]), and in differences in absolute risks by smoking and *NAT2* genotype. Smoking also interacted additively and positively with the GRS on any UC (*p* = 0.01) and non‐aggressive UC (*p* = 0.02), but not on aggressive UC (*p* = 0.1). Gene‐smoking interactions of lesser magnitude than for *NAT2* were found for SNPs in *APOBEC3A*, *SLC14A1* and *MYNN*.

**Conclusions:**

This study suggests that smoking increases UC risk more than expected when combined with certain genetic risks. Individuals with the slow acetylation *NAT2* variant might particularly benefit from smoking intervention to prevent lethal UC; however, replication in larger studies is needed.

## Graphical abstract

In this study, we found an interplay between smoking and genetic risk for BC in relation to UC risk, indicating that these factors jointly confer a greater UC risk than expected from the sum of their individual parts. We also found that smoking increased the risk of lethal UCs particularly in individuals with the slow acetylation genotype of NAT2 (rs1495741), suggesting that smoking prevention may be particularly important in this subgroup.

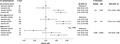

## 1 INTRODUCTION

Urothelial carcinoma is the most common histologic subtype of cancer of the urinary tract. Ninety‐five per cent of the cancers are located in the bladder, the most investigated site in etiological studies of urothelial cancer (UC). Tobacco smoking is the most important modifiable risk factor for the disease; the risk of bladder cancer (BC) for current smokers is around threefold higher than that of never smokers, and, depending on geographical region, sex and the ever‐changing smoking prevalence, smoking is estimated to cause around one third or more of all BCs.^1^, ^2^ Moreover, among BC patients, smoking has been associated with increased recurrence after transurethral resection of the bladder,^3^ and with poorer survival after radical cystectomy.^4^

The N‐acetyltransferase‐2 (*NAT2*) gene encodes for an enzyme that detoxifies a spectrum of carcinogens, including aromatic amines from tobacco smoke, which are thought to be particularly potent for the development of BC.^5^, ^6^, ^7^, ^8^ As expected based on biological plausibility, smoking has been shown to be a stronger risk factor for urothelial BC in individuals with the slow acetylation *NAT2* genotype than the intermediate or fast acetylation genotypes,^6^, ^9^ with strong *NAT2*‐smoking interactions on both the additive and multiplicative scale.^7^ Furthermore, when *NAT2* and another 11 single nucleotide polymorphisms (SNPs) predisposing to BC were combined in a genetic risk score (GRS), smoking and the GRS interacted positively on BC risk, with the combined risk exceeding the expected risk from the two risk factors individually (additive interaction).^7^ These findings suggest that smoking cessation for the prevention of BC may be particularly important in individuals with a certain genetic makeup. However, the implementation of such targeted prevention will benefit from knowledge of the interactive effects of genes and smoking on UC aggressiveness and fatality.

In this study, we investigated interactions between smoking and 18 SNPs including in the *NAT2* gene, individually and combined in a GRS, on UC risk overall and by disease aggressiveness including lethal outcome.

## 2 MATERIALS AND METHODS

### 2.1 Study population

The study population comprised 28,098 participants in the Malmö Diet and Cancer Study (MDCS) included in the European Prospective Investigation into Cancer and Nutrition (EPIC). The MDCS is a population‐based cohort in which participants undertook a health examination in 1991–96 at the ages of 44 to 73 years.^10^ Information on smoking was collected through a questionnaire that acquired details about smoking status, duration and smoking type (cigarettes, cigars/cigarillos and pipe) and dose in current smokers. A blood sample was drawn and separated before being stored at −80°C. In the present study, we included 25,453 individuals with complete smoking and genetic information without a prevalent cancer at baseline (*n* exclusions = 8, 1030 and 1607 respectively). Written informed consent was collected from all participants during study enrolment.

### 2.2 Genotyping and SNP selection

Genotyping was performed using the Illumina GSA v1 genotyping array. An internal quality control excluded samples with a low call rate (<90%), were out of Hardy–Weinberg equilibrium, or that exhibited discordance between reported and genetically inferred sex.^11^ The Haplotype Reference Consortium, a large reference panel of human haplotypes, was used to perform the genotype imputation.^12^

We included genetic variants associated with BC in populations of European ancestry in genome‐wide association (GWA) studies, 18 SNPs in total^13^, ^14^ (Table S1). A weighted GRS was created by multiplying each SNP, coded 0/1/2 to represent the number of risk alleles present, with the beta‐coefficient (or natural log odds ratio) from the association with BC in GWA studies. The individual SNP scores were summed into a GRS, which was composed of small effects by all individual SNPs rather than by strong effects of one or a few SNPs.

### 2.3 Case identification and classification

Using the unique personal identity number for each resident in Sweden, study participants were followed through 2018 in the nation‐wide Swedish Cancer Register, Cause of Death Register and the Total Population Register, which provide information on cancer diagnoses, death and its cause and emigration. Primary urothelial tract cancers, including carcinoma in situ, were ascertained with international classification of diseases (ICD) edition 7 codes 180.1 and 181 (ICD‐10 C65‐68 and D09 [0–1]) in the cancer register. Histopathological re‐evaluation of tumours was performed by an experienced pathologist (K. J.) and only verified UCs with sufficient tumour specimen from TURBT or cystectomy were included as events in the study. The 2004 WHO grading system was used for tumour classification. Non‐muscle invasive tumours (pTa, CIS and pT1) that had not led to death within the first 10 years of diagnosis were classified as non‐aggressive, and muscle invasive tumours (pT2–pT4) and UCs registered as the underlying cause of death within the first 10 years of diagnosis were classified as aggressive. We initially considered including only pT1 grade 1 and 2 in the non‐aggressive group; however, we opted for muscle invasiveness as the base for classification because these groups showed a greater difference in association with smoking and the GRS (Table S2) and with overall and UC‐specific survival (Figure S1). The addition of UC deaths to aggressive disease, which is commonly practiced in etiological studies of prostate cancer (e.g. reference no. 15), added statistical power to this subgroup without compromising on the heterogeneity in hazard ratios (HRs) according to disease aggressiveness.

### 2.4 Statistical analysis

We used Cox regression with age as the time‐scale to calculate HRs and 95% confidence intervals (CIs) of UC by smoking, the GRS, *NAT2* rs1495741, and by smoking jointly with the GRS and individual SNPs. Person‐years were counted from baseline until the date of UC diagnosis, death, emigration, or end of follow‐up, whichever occurred first. We adjusted the analyses for sex and education (six categories and missing [0.2%]). Due to evidence of an interaction between sex and the GRS in relation to all and non‐aggressive UC, we included a product term of sex and the GRS in these analyses. Schoenfeld residuals tests confirmed the proportional hazards assumption. The heterogeneity of the HRs between non‐aggressive and aggressive UC was calculated using the Lunn and McNeil duplication method.^16^

Interactions between smoking and the GRS and individual genetic variants were assessed with smoking and the genetic component as binary variables: never/ever smoker, </≥median GRS and homozygous risk variant/other for individual SNPs. When the homozygous risk variant of a SNP made up <20% of the study population, a recessive effects model was used instead. Multiplicative interaction was calculated using the Wald test of the product term of smoking and the genetic component in the Cox regression model. Additive interaction was calculated as the relative excess risk of interaction (RERI) expressed as RR11 − RR10 − RR01 + 1, with RR* representing the HR of: ever smokers with high genetic risk (RR11), ever smokers with low genetic risk (RR10) and never smokers with high genetic risk (RR01), compared to never smokers with low genetic risk (the reference group, “+1”). CIs and *p*‐values were obtained using the delta method by Hosmer and Lemeshow,^17^ calculated using Stata scripts provided by VanderWeele and Knol.^18^

Absolute risks of UC between 60 and 80 years of age were calculated as described by Gail et al.^19^ In this analysis, risks of UC and death as competing events were calculated in strata of 60–70 and 70–80 years of age. Because the incidence of UC is much higher among men than women, and there were relatively few UC cases in women in our study, we only calculated absolute risks for men.

All statistical analyses were performed in Stata 17 (StataCorp LLC).

## 3 RESULTS

### 3.1 Baseline characteristics and follow‐up

Out of the 25,453 individuals in the study, 7232 (28%) were current smokers and 8632 (34%) were ex‐smokers at baseline (Table 1). During an average follow‐up of 21 years, 520 individuals were diagnosed with UC, 339 of whom were categorised with non‐aggressive disease and 163 with aggressive disease.

**TABLE 1:** Characteristics of the 25,453 participants in the Malmö Diet and Cancer Study

	Men (*n* = 10,201)	Women (*n* = 15,252)	Total (*n* = 25,453)
Baseline age, years, mean (SD)	57.2 (7.9)	59.0 (7.0)	57.9 (7.6)
Categories, *n* (%)			
<55	3478 (34)	6923 (45)	10,401 (41)
55–64	4413 (43)	5349 (35)	9762 (38)
≥65	2310 (23)	2980 (20)	5290 (21)
Baseline smoking status, *n* (%)			
Never smoker	2881 (28)	6708 (44)	9589 (38)
Ex‐smoker	4391 (43)	4241 (28)	8632 (34)
Current smoker	2929 (29)	4303 (28)	7232 (28)
Education, *n* (%)^a^			
Incomplete elementary school	80 (1)	117 (1)	197 (1)
6–8 years (elementary school)	4586 (45)	5835 (38)	10,421 (41)
9–10 years (elementary school)	1997 (20)	4644 (31)	6641 (26)
11–12 years (high school)	1213 (12)	1068 (7)	2281 (9)
≥1 year after high school	940 (9)	1275 (8)	2215 (9)
University degree	1362 (13)	2276 (15)	3638 (14)
Years of follow‐up, mean (SD)	20.0 (6.9)	21.9 (5.4)	21.1 (6.1)
Categories, *n* (%)			
<5	483 (5)	304 (2)	787 (3)
5–14.9	1891 (19)	1584 (10)	3475 (14)
≥15	7827 (77)	13,364 (88)	21,191 (83)
Incident urothelial cancer, *n*	372	148	520
Non‐aggressive, *n* ^b^	239	100	339
Aggressive, *n* ^b^	123	40	163

Abbreviation: SD, standard deviation.

^a^ Information on education was missing in 60 (0.2%) individuals.

^b^ Non‐aggressive tumours included non‐muscle invasive (Ta, Tis and T1) tumours and aggressive tumours included muscle invasive (T2–T4) tumours and urothelial cancers recorded as the primary cause of death within 10 years of diagnosis.

### 3.2 Association of smoking, the GRS and *NAT2* with UC risk

Table 2 shows the results from main effects analyses of smoking, the GRS, and *NAT2* on UC risk. The HR of any UC was 3.73 (95% CI, 2.91–4.79) for current versus never smoking with no difference according to sex (*p*_sex‐interaction_ = 0.2) or disease aggressiveness (*p*_heterogeneity_ = 0.7), and the HR was 2.78 (95% CI, 2.21–3.50) for ever versus never smoking. The association between the GRS and UC was stronger for non‐aggressive UC (HR for top vs. bottom tertile, 3.40 [95% CI, 2.00–5.77]) than for aggressive UC (HR, 1.12 [95% CI, 0.77–1.63], *p*_heterogeneity_ = 0.049). The HR for the slow (GG) versus intermediate or fast (AA/AG) acetylation genotypes of *NAT2* was 1.10 (95% CI, 0.92–1.32) for any UC, with no difference according to sex (*p*_sex‐interaction_ = 0.7) or disease aggressiveness (*p*_heterogeneity_ = 0.3).

**TABLE 2:** Hazard ratio (95% confidence interval)^a^ of urothelial cancer in 25,453 men and women in the Malmö Diet and Cancer Study by smoking, *NAT2* rs1495741 and a bladder cancer genetic risk score

	All urothelial cancer						*N*_cases_	Non‐aggressive UC^b^	*N*_cases_	Aggressive UC^b^
	*N*_cases_	Men	*N*_cases_	Women	*N*_cases_	Total				
Never smoker	49	Reference	42	Reference	91	Reference	59	Reference	27	Reference
Ever smoker	323	3.02 (2.23–4.08)	106	2.44 (1.70–3.50)	429	2.78 (2.21–3.50)	280	2.77 (2.09–3.69)	136	2.95 (1.94–4.49)
		*p*_sex‐interaction_ = 0.4^c^						*p*_sex‐interaction_ = 0.9		*p*_sex‐interaction_ = 0.2
								*p*_heterogeneity_ = 0.7^d^		
Never smoker	49	Reference	42	Reference	91	Reference	59	Reference	27	Reference
Ex‐smoker	171	2.42 (1.76–3.32)	46	1.97 (1.29–2.99)	217	2.21 (1.73–2.84)	144	2.28 (1.67–3.09)	67	2.24 (1.42–3.52)
Current smoker	152	4.24 (3.06–5.86)	60	3.03 (2.03–4.52)	212	3.73 (2.91–4.79)	136	3.59 (2.63–4.89)	69	4.24 (2.70–6.65)
		*p*_sex‐interaction_ = 0.2^c^						*p*_sex‐interaction_ = 0.4^c^		*p*_sex‐interaction_ = 0.6^c^
								*p*_heterogeneity_ = 0.7^d^		
Never smoker	49	Reference	42	Reference	91	Reference	59	Reference	27	Reference
Ex‐smoker	171	2.42 (1.76–3.32)	46	1.97 (1.29–2.99)	171	2.22 (1.73–2.85)	144	2.27 (1.67–3.08)	67	2.27 (1.44–3.57)
Smoker <median packy^e^	52	4.31 (2.91–6.38)	31	2.67 (1.67–4.27)	52	3.54 (2.62–4.77)	58	3.67 (2.55–5.29)	22	3.38 (1.92–5.95)
Smoker ≥median packy^e^	95	4.31 (3.04–6.10)	27	3.70 (2.27–6.03)	95	4.00 (3.03–5.27)	74	3.64 (2.57–5.15)	44	4.96 (3.04–8.08)
		*p*_sex‐interaction_ = 0.5^c^						*p*_sex‐interaction_ = 0.3^c^		*p*_sex‐interaction_ = 0.7^c^
								*p*_heterogeneity_ = 0.4^d^		
GRS <median	158	Reference	54	Reference	212	Reference	125	Reference	75	Reference
GRS ≥median	214	1.36 (1.11–1.68)	94	1.75 (1.25–2.44)	308	1.75 (1.25–2.44)	214	2.46 (1.60–3.79)	88	1.18 (0.87–1.60)
		*p*_sex‐interaction_ = 0.2^c^						*p*_sex‐interaction_ = 0.05^c^		*p*_sex‐interaction_ = 0.5^c^
								*p*_heterogeneity_ = 0.051^d^		
GRS tertile 1	108	Reference	33	Reference	141	Reference	66	Reference	52	Reference
GRS tertile 2	117	1.07 (0.83–1.40)	46	1.42 (0.91–2.22)	163	1.34 (1.00–1.78)	71	1.73 (1.18–2.53)	54	1.03 (0.70–1.51)
GRS tertile 3	147	1.40 (1.09–1.79)	69	2.11 (1.40–3.20)	216	2.08 (1.39–3.12)	102	3.40 (2.00–5.77)	57	1.12 (0.77–1.63)
		*p*_sex‐interaction_ = 0.1^c^						*p*_sex‐interaction_ = 0.02^c^ ^,^ ^f^		*p*_sex‐interaction_ = 0.6^c^
								*p*_heterogeneity_ = 0.049^d^		
*NAT2* rs1495741 AA/AG	136	Reference	56	Reference	192	Reference	94	Reference	55	Reference
*NAT2* rs1495741 GG	236	1.12 (0.91–1.39)	92	1.05 (0.75–1.47)	328	1.10 (0.92–1.32)	145	1.05 (0.84–1.31)	108	1.26 (0.91–1.75)
		*p*_sex‐interaction_ = 0.7^c^						*p*_sex‐interaction_ = 0.5^c^		*p*_sex‐interaction_ = 0.2^c^
								*p*_heterogeneity_ = 0.3^d^		

Abbreviations: GRS, genetic risk score; packy, pack‐years; UC, urothelial cancer.

^a^ Hazard ratios were calculated by use of Cox regression with attained age as time‐scale, adjusted for education level (six categories and missing [0.2%]), sex (in the full‐population analysis) and a product term of sex and the genetic risk score in the analyses of the genetic risk score in relation to all urothelial cancer and non‐aggressive disease.

^b^ Non‐aggressive tumours included non‐muscle invasive (Ta, Tis and T1) tumours and aggressive tumours included muscle invasive (T2–T4) tumours and urothelial cancers recorded as the primary cause of death within 10 years of diagnosis.

^c^ The *p*‐value for sex‐interaction was calculated with the Wald test of the product between sex and the exposure as an ordinal variable.

^d^ The *p*‐value for heterogeneity in hazard ratios for non‐aggressive versus aggressive disease was calculated using the Lunn and McNeil test.^16^

^e^ The median pack‐years among smokers was 23. The information was missing in 311 smokers (173 men and 138 women).

^f^ Hazard ratio, tertile 2 and 3: men, 1.07 (0.76–1.49), 1.58 (1.16–2.15); women, 2.40 (1.28–4.49), 3.83 (2.13–6.90).

### 3.3 Combined association of smoking status and the GRS with UC risk

The combined association of smoking status (never, ex‐, or current smoker) and the GRS in tertiles with risk of any UC showed a stepwise increased risk by increasing levels of each of the two risk factors, including for higher GRS among never smokers (Table 3). Current smokers in the top tertile of the GRS had a HR for any UC of 7.52 (95% CI, 4.39–12.9) compared to never smokers in the lowest GRS tertile.

**TABLE 3:** Hazard ratio of all urothelial cancer by the combination of smoking status and tertiles of a bladder cancer genetic risk score in 25,453 men and women in the Malmö Diet and Cancer Study

Smoking status	GRS tertile 1		GRS tertile 2		GRS tertile 3	
	*N*_cases_	HR (95% CI)^a^	*N*_cases_	HR (95% CI)^a^	*N*_cases_	HR (95% CI)^a^
Never smoker	26	Reference	27	1.19 (0.68–2.08)	38	1.89 (1.06–3.37)
Ex‐smoker	58	2.08 (1.30–3.32)	71	2.78 (1.73–4.47)	88	4.15 (2.40–7.18)
Current smoker	57	3.27 (2.05–5.21)	65	4.68 (2.89–7.57)	90	7.52 (4.39–12.9)

Abbreviations: CI, confidence interval; GRS, genetic risk score; HR, hazard ratio.

^a^ Hazard ratios were calculated by use of Cox regression with attained age as time‐scale, adjusted for education level (six categories and missing [0.2%]), sex and a product term of sex and the genetic risk score.

### 3.4 Gene‐smoking interactions and UC risk

**FIGURE 1:**
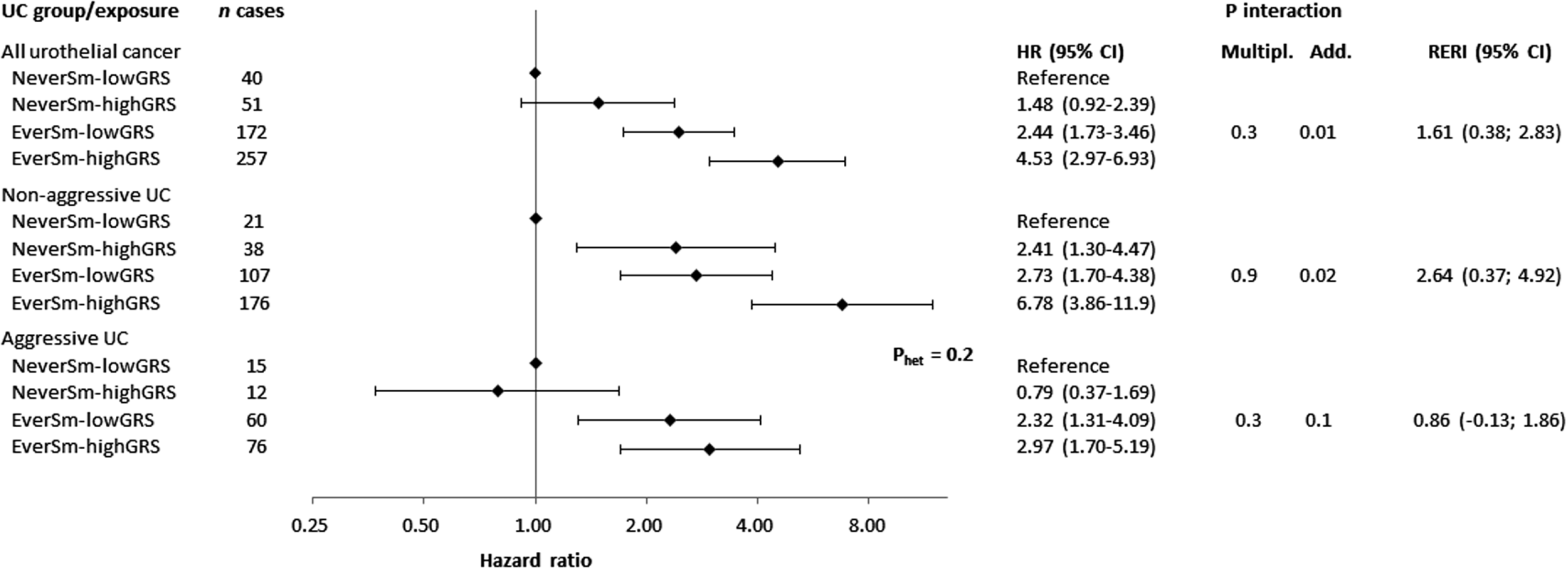
Hazard ratio (95% confidence interval) of urothelial cancer (UC), and results from multiplicative and additive interaction tests, of smoking (never smoker [NeverSm] or ever smoker [EverSm]) and a bladder cancer genetic risk score (lowGRS <median or highGRS ≥median) combined, in 25,453 men and women in the Malmö Diet and Cancer Study. Hazard ratios were calculated by use of Cox regression with attained age as time‐scale, adjusted for education level (six categories and missing [0.2%]), sex and a product term of sex and the genetic risk score. The *p*‐value for multiplicative interaction was calculated with the Wald test of a 2 × 2 product of smoking and the genetic risk score. The *p*‐value and the relative excess risk of interaction (RERI) was calculated as described by VanderWeele and Knol.^18^ Non‐aggressive tumours included non‐muscle invasive (Ta, Tis and T1) tumours and aggressive tumours included muscle invasive (T2–T4) tumours and urothelial cancers recorded as the primary cause of death within 10 years of diagnosis. The *p*‐value for heterogeneity (*p*_het_) in hazard ratios for non‐aggressive versus aggressive disease was calculated using the Lunn and McNeil test^16^

**FIGURE 2:**
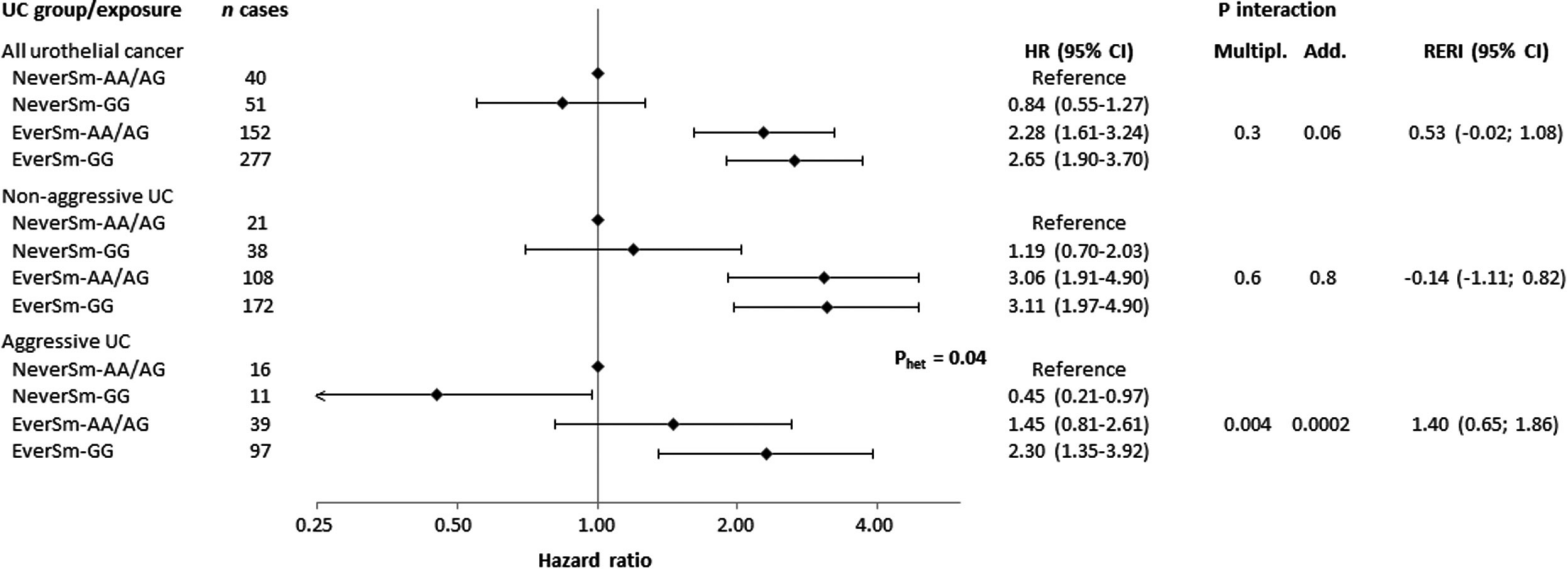
Hazard ratio (95% confidence interval) of urothelial cancer (UC), and results from multiplicative and additive interaction tests, of smoking (never smoker [NeverSm] or ever smoker [EverSm]) and *NAT2* rs1495741 (GG/AG or AA) combined, in 25,453 men and women in the Malmö Diet and Cancer Study. Hazard ratios were calculated by use of Cox regression with attained age as time‐scale, adjusted for education level (six categories and missing [0.2%]), sex and a product term of sex and the genetic risk score. The *p*‐value for multiplicative interaction was calculated with the Wald test of a 2 × 2 product of smoking and the genetic risk score. The *p*‐value and the relative excess risk of interaction (RERI) was calculated as described by VanderWeele and Knol.^18^ Non‐aggressive tumours included non‐muscle invasive (Ta, Tis and T1) tumours and aggressive tumours included muscle invasive (T2–T4) tumours and urothelial cancers recorded as the primary cause of death within 10 years of diagnosis. The *p*‐value for heterogeneity (*p*_het_) in hazard ratios for non‐aggressive versus aggressive disease was calculated using the Lunn and McNeil test^16^

Two‐by‐two interaction tests of smoking with the GRS and individual SNPs showed interactions for any, aggressive, or non‐aggressive UC at *p* < 0.05 for the GRS, *NAT2* rs1495741, *APOBEC3A* rs1014971, *SLC14A1* rs10775480 and *MYNN* rs10936599 (Table S3). Smoking and the GRS interacted additively and positively in relation to any UC (*p*_additive_ = 0.01) and non‐aggressive UC (*p*_additive_ = 0.02), but not with aggressive UC (*p*_additive_ = 0.1) (Figure 1). The strongest gene‐smoking interaction observed was for *NAT2* in relation to aggressive UC (*p*_additive_ = 0.0002, *p*_multiplicative_ = 0.004). This manifested in a greater HR for ever smoking among individuals with the slow acetylation genotype (HR, 5.00 [95% CI, 2.67–9.38]) than with the intermediate or fast genotypes (HR, 1.50 [95% CI, 0.83–2.71]). This pattern was not observed for non‐aggressive UC, as depicted by a similar HR for ever versus never smoking for the slow and the intermediate/fast acetylation genotypes (Table 4), and overall differential HRs by disease aggressiveness for smoking and *NAT2* combined (*p*_heterogeneity_ = 0.04, Figure 2). Interactions between smoking and *APOBEC3A* , *SLC14A1* and *MYNN*, respectively, were of lesser magnitude than for *NAT2*, and were similar for non‐aggressive and aggressive UC (Figures S2–S4).

**TABLE 4:** Hazard ratio (95% confidence interval)^a^ of urothelial cancer by ever versus never smoking, in subgroups of *NAT2* rs1495741 genotype, in 25,453 men and women in the Malmö Diet and Cancer Study

*NAT2* genotype	All urothelial cancer	Non‐aggressive UC	Aggressive UC
*NAT2* rs1495741 AA/AG	2.28 (1.60–3.25)	3.02 (1.88–4.85)	1.50 (0.83–2.71)
*NAT2* rs1495741 GG	3.16 (2.34–4.28)	2.64 (1.85–3.77)	5.00 (2.67–9.38)

Abbreviation: UC, urothelial cancer.

^a^ Hazard ratios were calculated by use of Cox regression with attained age as time‐scale, adjusted for education level (six categories and missing [0.2%]) and sex. The number of cases in each *NAT2*‐smoking category is reported in Figure 2.

### 3.5 Absolute risks of UC by combinations of smoking with the GRS and *NAT2*

In 60‐year‐old men with a low GRS, the 20‐year risk of any UC was 1.5% in never smokers and 4.2% in ever smokers. The corresponding risks for men with a high GRS were 1.9% and 5.8% respectively. The 20‐year risk of aggressive UC for 60‐year‐old men with the intermediate or fast acetylation *NAT2* genotypes was 0.5% in never smokers and 1.3% in ever smokers. These risks were for men with the slow acetylation genotype 0.4% and 1.9% respectively.

## 4 DISCUSSION

In this prospective study, we found that smoking and the slow acetylation *NAT2* genotype in combination posed a greater risk for aggressive UC than expected based on the associations of these risk factors individually. Similarly, we found a higher risk of non‐aggressive UC than expected for the combination of smoking and high genetic risk (GRS). These findings further detail the gene–smoking interactions previously found for urothelial BC overall,^6^, ^7^ and provide further knowledge to form targeted smoking interventions.

*NAT2* is a regulatory gene of phase II detoxification of carcinogens from tobacco smoke, the most important modifiable risk factor for UC including BC. In tobacco smoke, aromatic amines are thought to be the most potent chemical compounds to cause BC,^8^ and the detoxification of aromatic amines regulated by *NAT2* could be the primary way through which *NAT2* is related to BC risk in smokers.^6^, ^7^, ^9^ Knowledge of the interaction between *NAT2* and smoking on overall BC risk is not new.^6^, ^20^ However, investigation of an interaction on the additive scale has only been conducted more recently by Garcia‐Closas et al.^7^ They found a stronger *NAT2*‐smoking interaction on the additive than on the multiplicative scale, just as we did, which more readily facilitates the identification of the group that will benefit most from intervention.^18^ Whilst the interaction on the multiplicative scale implies a difference in the relative risk of smoking according to *NAT2* acetylation, an interaction on the additive scale implies a difference in the absolute risk of smoking according to *NAT2* acetylation. This information is needed to estimate the absolute number of preventable cases among smokers. In our study, we provide absolute risk estimates for men, which warrant replication and precision through the use of a larger sample. Furthermore, for a smoking intervention according to *NAT2* genotype to be cost‐efficient and adequately motivating for the individual, evidence of an interaction will also be needed in relation to more common smoking‐related diseases, or in relation to UC prognosis in patients after smoking cessation. It would thus be relevant to investigate these areas in response to our novel findings, specifically for aggressive UC.

Around one third of BCs have been estimated to be caused by heritable factors.^21^, ^22^ Only a few rare mutations with strong effects on BC have been identified to explain the increased genetic risk, therefore the BC risk attributed to genetics is assumed to be polygenic, composed of small effects by multiple gene loci.^23^ Two of these, *NAT2* and *GSTM1* (another carcinogen detoxifying gene), were identified through a candidate gene approach, and additional BC variants, primarily with unknown functions, were detected in later GWA studies.^13^, ^14^ In our study, we had data on 10 of the 12 genetic variants previously investigated by Garcia‐Closas et al on total BC,^7^ excluding variants in *GSTM1* and *UGT1A6*. We (i) replicated an additive interaction between *APOBEC3A*‐rs1014971 and smoking on total UC with similar findings according to UC aggressiveness, (ii) found a negative interaction between smoking and *SLC14A1*‐rs10775480, in contrast to the finding by Garcia‐Closas et al, and (iii) among eight additional SNPs investigated in our study, we found a positive and additive interaction between smoking and *MYNN*‐rs10936599 on total and non‐aggressive UC. When combining all genetic variants into a GRS, we observed a stronger main effect on non‐aggressive UC, as expected due to the dominance of non‐aggressive tumours in GWA studies. We also replicated the additive GRS‐smoking interaction on total UC/BC observed by Garcia‐Closas et al.^7^ In our study, the interaction was stronger for non‐aggressive UC than for aggressive UC, possibly due to the lower statistical power for aggressive UC. Taken together, these findings suggest the potential for targeted smoking intervention based on genetic risk, which should be evaluated alongside gene‐smoking interactions for other major smoking‐related diseases.

The main limitation of our study is the relatively small population size and few cases of aggressive UC. As a result, the strong interaction found for smoking and *NAT2* in aggressive disease could be a chance finding. However, the lack of interaction in the non‐aggressive group, where we had greater statistical power, supports the possibility that aggressive disease drives the previously found robust *NAT2*‐smoking interaction on total urothelial BC.^6^, ^7^ Yet, further gene‐smoking interactions could exist for aggressive UC that have gone undetected in our study. We also lacked information on occupational exposures to carcinogens, which could exhibit a dual effect on our results through an association with smoking habits, and through genetic susceptibility to detoxify carcinogens such as work‐exposed aromatic amines.^5^, ^8^ However, in a population‐based study such as ours, occupational exposure to UC carcinogens is rare, and is therefore expected to have a limited effect on the results. We also lacked repeat smoking information during follow‐up, which could have been used for optimised smoking exposure in a time‐updated analysis. The baseline smoking information in our study is, however, of expected good validity, supported by the similar effect estimates for baseline smoking on risk in our study as compared to in a meta‐analysis,^1^ and the observed dose–response effect for smoking status and pack‐years on risk in our study. Other main strengths of our study include the complete follow‐up of participants through individual tracking in Swedish registers,^24^ and the inclusion of only histopathological verified UC cases evaluated by a single experienced pathologist.

In conclusion, this prospective study substantiates gene‐smoking interactions on overall UC risk, and further specifies the additive interactions between smoking and (i) *NAT2* on aggressive UC, and (ii) a GRS on non‐aggressive UC, suggesting a higher‐than‐expected UC risk for these gene‐smoking combinations. These findings reveal the potential for smoking interventions targeting individuals with high genetic risk, such as individuals with the slow acetylation *NAT2* variant to prevent lethal UC. These findings require replication in larger studies, and because UC is a relatively rare disease, the findings should be evaluated alongside gene‐smoking interactions for other major smoking‐related diseases.

## CONFLICT OF INTEREST

The authors made no disclosures.

## AUTHOR CONTRIBUTIONS

Stanley Teleka was involved in conceptualization, data curation, investigation and writing–review and editing; Sylvia H. J. Jochems was involved in investigation and writing–review and editing; Karin Jirström was involved in data curation, investigation and writing–review and editing; Tanja Stocks was involved in conceptualization, data curation, formal analysis, funding acquisition, investigation and writing–original draft.

## ETHICS STATEMENT

The study was approved by the ethics committee at Lund University, Sweden (no. 2014/830).

## Supporting information


Table S1
Table S2
Table S3
Figure S1
Figure S2
Figure S3
Figure S4

## Data Availability

The data that support the findings of this study are available from https://www.malmo‐kohorter.lu.se with ethical approval and permission from the steering committee. Restrictions apply to the availability of the data, appropriate procedures were followed to obtain data for this study.
