# Supplementary material for: The interaction between smoking and bladder cancer genetic variants on urothelial cancer risk by disease aggressiveness
Source: Cancer Med. 2022 Mar 13;11(15):2896–905. doi: 10.1002/cam4.4654 (PMC9359879; doi:10.1002/cam4.4654)
Supplement: Supplementary file 1 — Table S1 Table S2 Table S3 Figure S1 Figure S2 Figure S3 Figure S4 [file CAM4-11-2896-s001.docx]

**Supplementary material**

**The interaction between smoking and bladder cancer genetic variants on urothelial cancer risk by disease aggressiveness**

Stanley Teleka, Sylvia HJ Jochems, Karin Jirström, Tanja Stocks

Supplementary Table 1. Bladder cancer genetic variants investigated in the study

| **Gene (or nearby), SNP** | **Risk allele** | **Other allele** | **Risk allele frequency^a^** | **Odds ratio per risk allele^b^** |
| --- | --- | --- | --- | --- |
| *NAT2*, rs1495741 | A | G | 0.78 | 1.13 |
| APOBEC3A, rs1014971 | T | C | 0.63 | 1.18 |
| *SLC14A1*, rs10775480 | T | C | 0.44 | 1.13 |
| *MYNN*, rs10936599 | C | T | 0.75 | 1.15 |
| *UGT1A*, rs11892031 | A | C | 0.91 | 1.17 |
| *SLC14A1*, rs17674580 | T | C | 0.35 | 1.17 |
| *PSCA*, rs2294008 | T | C | 0.46 | 1.13 |
| *TERT*, rs2736098 | A | G | 0.27 | 1.16 |
| *CLPTM1L*, rs401681 | C | T | 0.55 | 1.12 |
| *MCF2L*, rs4907479 | A | G | 0.27 | 1.13 |
| *LINC02871*, rs6104690 | A | G | 0.53 | 1.07 |
| *JAG1*, rs62185668 | A | C | 0.24 | 1.19 |
| *TP63*, rs710521 | A | G | 0.75 | 1.18 |
| *SLC14A1*, rs7238033 | C | T | 0.56 | 1.20 |
| *TACC3*, rs798766 | T | C | 0.21 | 1.20 |
| *CCNE1*, rs8102137 | C | T | 0.33 | 1.13 |
| *LSP1*, rs907611 | A | G | 0.34 | 1.15 |
| *MYC*, rs9642880 | T | G | 0.50 | 1.21 |

^a^In the study population.

^b^From prior genome-wide association studies (references no. 13 and 14 in the article).

Supplementary Table 2. Hazard ratio of urothelial cancer using different categorisations of urothelial cancer aggressiveness based on pathological stage and grade, by smoking status and genetic risk score

|  | **Our used categorisation^a^** | | | | **Alternative categorisation** | | | |
| --- | --- | --- | --- | --- | --- | --- | --- | --- |
|  | **pTa, CIS and pT1** | | **pT2-pT4** | | **pTa grade 1 and 2** | | **CIS, pT1-pT4, or grade 3** | |
|  | **N_cases_** | **HR (95% CI)^b^** | **N_cases_** | **HR (95% CI)^b^** | **N_cases_** | **HR (95% CI)^b^** | **N_cases_** | **HR (95% CI)^b^** |
| Never smoker | 68 | Reference | 18 | Reference | 33 | Reference | 53 | Reference |
| Ever smoker | 306 | 2.62 (2.01-3.42) | 110 | 3.65 (2.20-6.04) | 152 | 2.68 (1.83-3.93) | 264 | 2.92 (2.17-3.94) |
|  | P_heterogeneity_=0.2^c^ | | | | P_heterogeneity_=0.6^c^ | | | |
| GRS <median | 139 | Reference | 61 | Reference | 67 | Reference | 133 | Reference |
| GRS ≥median | 235 | 2.28 (1.52-3.44) | 67 | 1.10 (0.78-1.56) | 118 | 2.58 (1.45-4.59) | 184 | 1.52 (0.98-2.36) |
|  | P_heterogeneity_=0.04^c^ | | | | P_heterogeneity_=0.2^c^ | | | |

GRS, genetic risk score; HR, hazard ratio; CI, confidence interval.

^a^This categorisation was used in the present study for non-aggressive and aggressive urothelial cancer, respectively, with the addition of cases recorded with death from urothelial cancer within 10 years of diagnosis to aggressive disease.

^b^Hazard ratios were calculated by use of Cox regression with attained age as time-scale, adjusted for education level (six categories and missing [0.2%]), sex, and a product term of sex and the genetic risk score in the analyses of the genetic risk score in relation to the lower stage categories (pTa/CIS/pT1 and pTa grade 1-2), similar to the analysis of non-aggressive urothelial cancer in Table 2.

^c^The P-value for heterogeneity in hazard ratios for subgroups by stage and grade was calculated using the Lunn and McNeil test (reference no. 16 in the article).

Supplementary Table 3. P-values^a^ for the interaction between smoking and a bladder cancer genetic risk score and its individual SNPs in 25,453 men and women in the Malmö Diet and Cancer Study.

|  | **All urothelial cancer** | | **Non-aggressive UC^b^** | | **Aggressive UC^b^** | |
| --- | --- | --- | --- | --- | --- | --- |
| **Gene (or nearby), SNP** | **P_Mult._** | **P_Add._** | **P_Mult._** | **P_Add._** | **P_Mult._** | **P_Add._** |
| Genetic risk score | 0.3 | **0.01** | 0.9 | **0.02** | 0.3 | 0.1 |
| *NAT2*, rs1495741 | 0.2 | 0.06 | 0.6 | 0.8 | **0.004** | **0.0002** |
| [*APOBEC3A*](https://www.ebi.ac.uk/gwas/genes/APOBEC3A), rs1014971 | 0.3 | **0.04** | 0.5 | 0.09 | 0.3 | 0.2 |
| *SLC14A1*, rs10775480 | **0.003** | **0.045** | 0.08 | 0.3 | **0.03** | 0.1 |
| *MYNN*, rs10936599 | 0.05 | **0.01** | 0.3 | **0.04** | 0.09 | 0.2 |
| *UGT1A*, rs11892031 | 0.9 | 0.3 | 0.8 | 0.6 | 0.8 | 0.3 |
| *SLC14A1*, rs17674580 | 0.09 | 0.3 | 0.6 | 0.7 | 0.09 | 0.2 |
| *PSCA*, rs2294008 | 0.7 | 0.4 | 0.8 | 0.5 | 1.0 | 0.8 |
| *TERT*, rs2736098 | 0.8 | 0.3 | 0.4 | 0.8 | 0.2 | 0.4 |
| *CLPTM1L*, rs401681 | 0.3 | 0.8 | 0.5 | 1.0 | 0.1 | 0.5 |
| *MCF2L*, rs4907479 | 0.4 | 0.1 | 0.5 | 0.2 | 0.5 | 0.3 |
| *LINC02871*, rs6104690 | 0.3 | 0.2 | 0.3 | 0.3 | 0.4 | 0.5 |
| *JAG1*, rs62185668 | 0.4 | 0.3 | 0.4 | 0.3 | 0.6 | 0.5 |
| *TP63*, rs710521 | 0.2 | 0.7 | 0.09 | 0.5 | 0.6 | 0.9 |
| *SLC14A1*, rs7238033 | 0.5 | 0.7 | 0.9 | 0.6 | 0.2 | 0.2 |
| *TACC3*, rs798766 | 0.1 | 1.0 | 0.1 | 0.9 | 0.8 | 0.7 |
| *CCNE1*, rs8102137 | 0.7 | 0.6 | 0.4 | 0.7 | 0.4 | 0.4 |
| *LSP1*, rs907611 | 0.5 | 0.2 | 0.9 | 0.6 | 0.5 | 0.2 |
| *MYC*, rs9642880 | 0.4 | 0.2 | 0.7 | 0.2 | 0.4 | 0.3 |

^a^The p-value for multiplicative interaction represents the Wald test of the product of smoking (ever/never smoker) with the genetic risk score (</≥median) and each SNP, respectively, derived from Cox regression with attained age as time-scale with additional inclusion of the main associations of the risk allele and smoking, education level (six categories and missing [0.2%]), and sex. We assumed a dominant SNP effect, except when the homozygous risk variant made up less than 20%, in which case a recessive effect model was used (for rs17674580, rs2736098, rs4907479, rs62185668, rs798766, rs8102137, and rs907611, risk allele frequency≤0.35). The P-value for additive interaction was calculated as described by VanderWeele and Knol (reference no. 18 in the article).

^b^Non-aggressive tumors included non-muscle invasive (Ta, Tis, and T1) tumors and aggressive tumors included muscle-invasive (T2-T4) tumours and urothelial cancers recorded as the primary cause of death within 10 years of diagnosis.


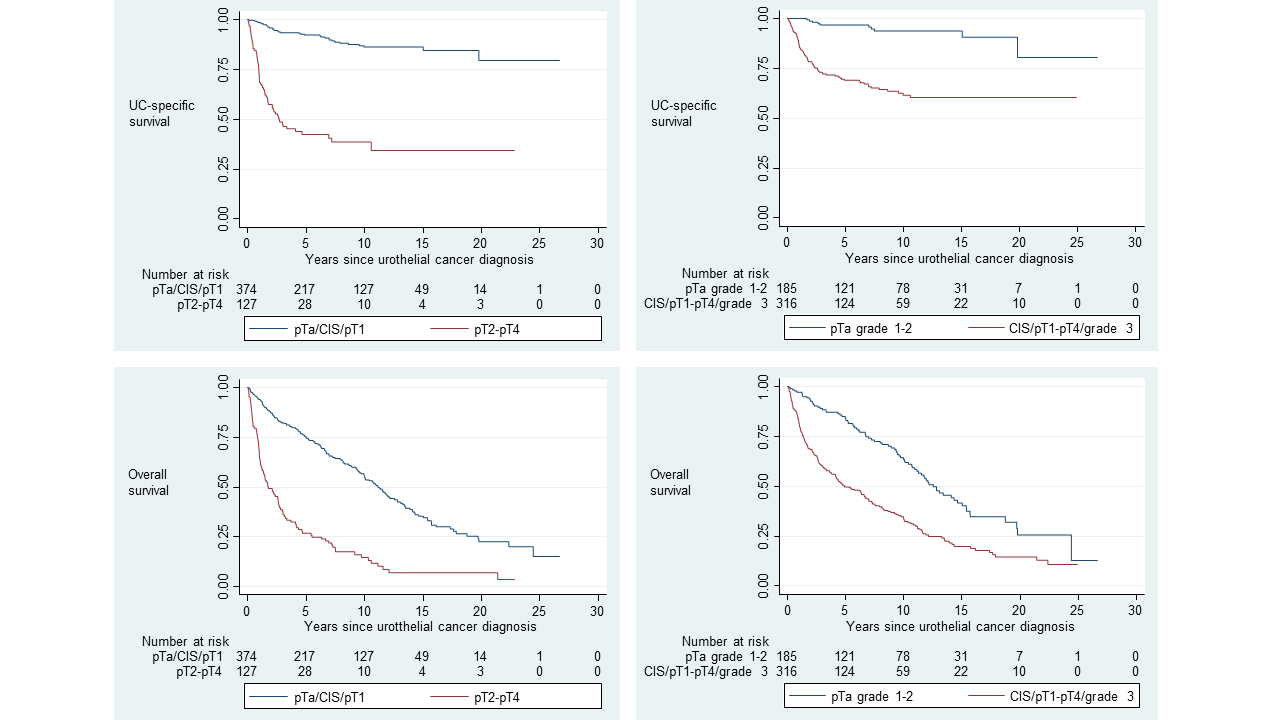


Supplementary Figure 1. Kaplan-Meier survival curves of urothelial cancer (UC)-specific and overall survival by various categorisations of urothelial cancer aggressiveness based on pathological stage and grade. The P-value for the log-rank test was <0.001 in all analyses.


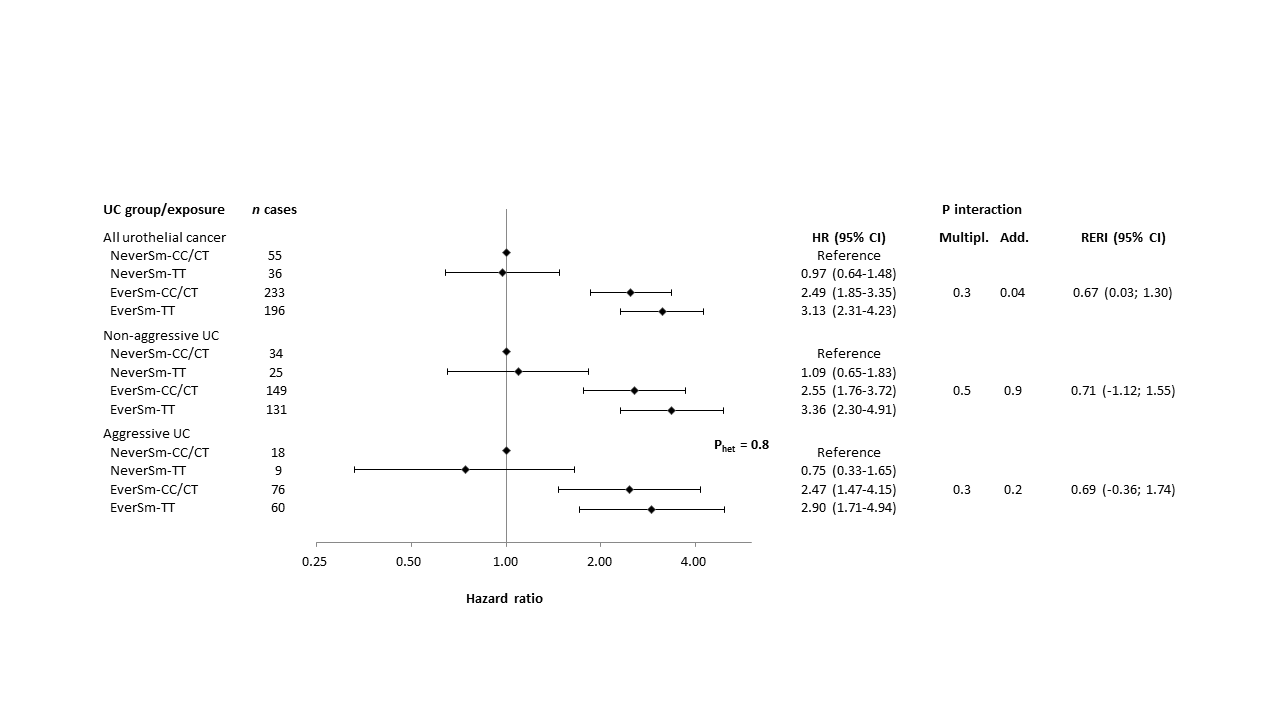


Supplementary Figure 2. Hazard ratio (95% confidence interval) of urothelial cancer (UC), and results from multiplicative and additive interaction tests, of smoking (never smoker [NeverSm] or ever smoker [EverSm]) and APOBEC3A rs1014971 (CC/CT or TT) combined, in 25,453 men and women in the Malmö Diet and Cancer Study. Hazard ratios were calculated by use of Cox regression with attained age as time-scale, adjusted for education level (six categories and missing [0.2%]), sex, and a product term of sex and the genetic risk score. The P-value for multiplicative interaction was calculated with the Wald test of a 2×2 product of smoking and the genetic risk score. The P-value and the relative excess risk of interaction (RERI) was calculated as described by VanderWeele and Knol (reference no. 18 in the article). Non-aggressive tumors included non-muscle invasive (Ta, Tis, and T1) tumors and aggressive tumors included muscle-invasive (T2-T4) tumours and urothelial cancers recorded as the primary cause of death within 10 years of diagnosis. The P-value for heterogeneity (P_het_) in hazard ratios for non-aggressive *vs* aggressive disease was calculated using the Lunn and McNeil test (reference no. 16 in the article).


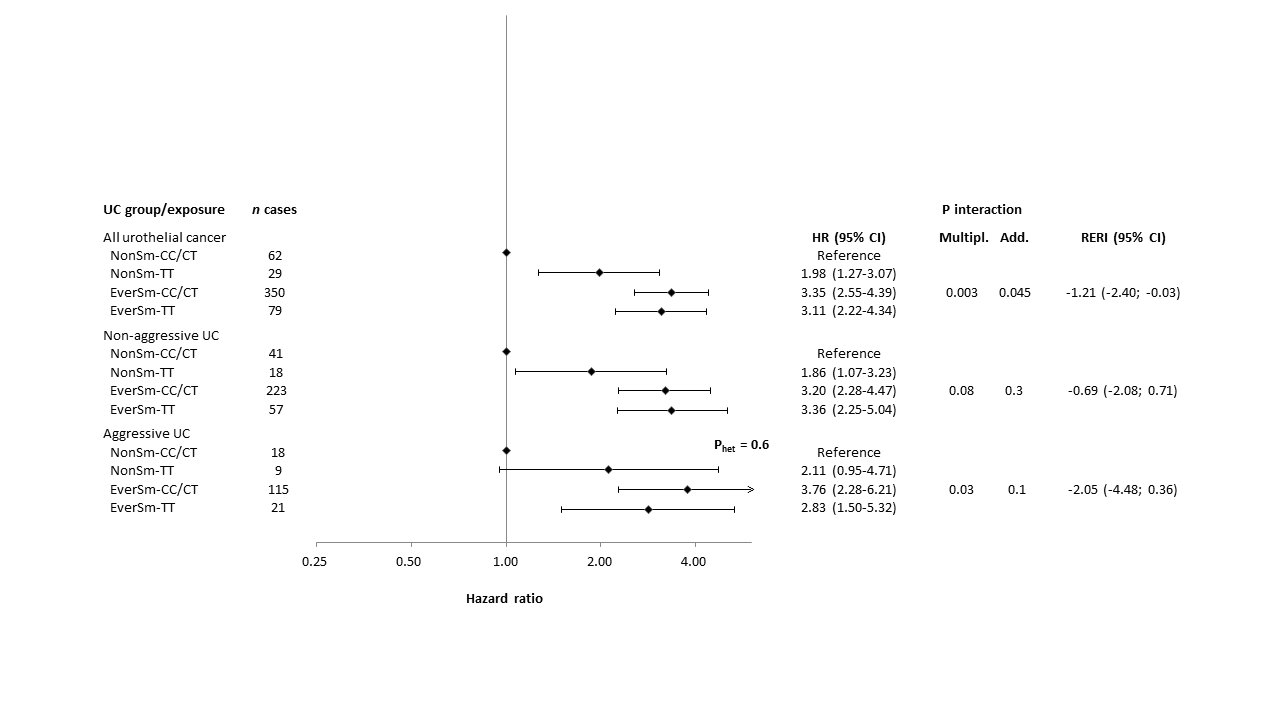


Supplementary Figure 3. Hazard ratio (95% confidence interval) of urothelial cancer (UC), and results from multiplicative and additive interaction tests, of smoking (never smoker [NeverSm] or ever smoker [EverSm]) and *SLC14A1* rs10775480 (CC/CT or TT) combined, in 25,453 men and women in the Malmö Diet and Cancer Study. Hazard ratios were calculated by use of Cox regression with attained age as time-scale, adjusted for education level (six categories and missing [0.2%]), sex, and a product term of sex and the genetic risk score. The P-value for multiplicative interaction was calculated with the Wald test of a 2×2 product of smoking and the genetic risk score. The P-value and the relative excess risk of interaction (RERI) was calculated as described by VanderWeele and Knol (reference no. 18 in the article). Non-aggressive tumors included non-muscle invasive (Ta, Tis, and T1) tumors and aggressive tumors included muscle-invasive (T2-T4) tumours and urothelial cancers recorded as the primary cause of death within 10 years of diagnosis. The P-value for heterogeneity (P_het_) in hazard ratios for non-aggressive *vs* aggressive disease was calculated using the Lunn and McNeil test (reference no. 16 in the article).


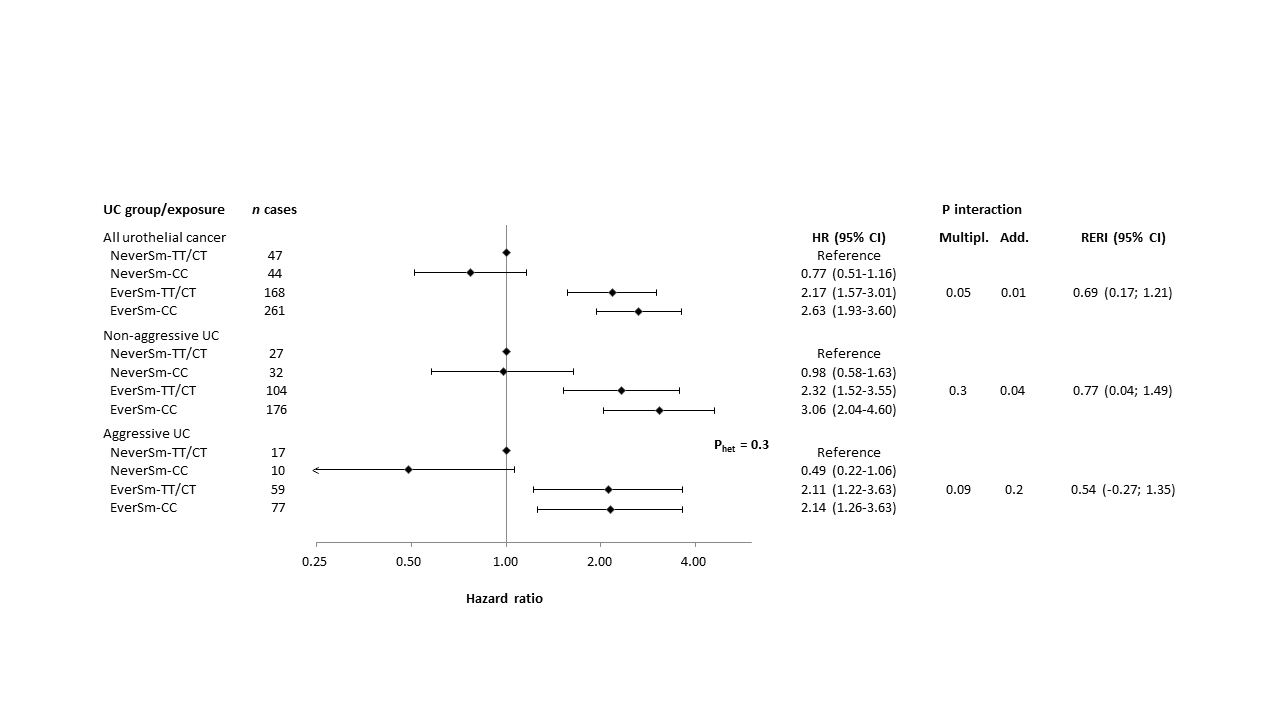


Supplementary Figure 4. Hazard ratio (95% confidence interval) of urothelial cancer (UC), and results from multiplicative and additive interaction tests, of smoking (never smoker [NeverSm] or ever smoker [EverSm]) and *MYNN* rs10936599 (TT/CT or CC) combined, in 25,453 men and women in the Malmö Diet and Cancer Study. Hazard ratios were calculated by use of Cox regression with attained age as time-scale, adjusted for education level (six categories and missing [0.2%]), sex, and a product term of sex and the genetic risk score. The P-value for multiplicative interaction was calculated with the Wald test of a 2×2 product of smoking and the genetic risk score. The P-value and the relative excess risk of interaction (RERI) was calculated as described by VanderWeele and Knol (reference no. 18 in the article). Non-aggressive tumors included non-muscle invasive (Ta, Tis, and T1) tumors and aggressive tumors included muscle-invasive (T2-T4) tumours and urothelial cancers recorded as the primary cause of death within 10 years of diagnosis. The P-value for heterogeneity (P_het_) in hazard ratios for non-aggressive *vs* aggressive disease was calculated using the Lunn and McNeil test (reference no. 16 in the article).
